# CDK4 inhibition diminishes p53 activation by MDM2 antagonists

**DOI:** 10.1038/s41419-018-0968-0

**Published:** 2018-09-11

**Authors:** Anusha Sriraman, Antje Dickmanns, Zeynab Najafova, Steven A. Johnsen, Matthias Dobbelstein

**Affiliations:** 1 Institute of Molecular Oncology, Göttingen Center of Molecular Biosciences (GZMB), University Medical Center Göttingen, D-37077 Göttingen, Germany; 2 Department of General, Visceral, and Pediatric Surgery, University Medical Center Göttingen, D-37077 Göttingen, Germany

## Abstract

The genes encoding MDM2 and CDK4 are frequently co-amplified in sarcomas, and inhibitors to both targets are approved or clinically tested for therapy. However, we show that inhibitors of MDM2 and CDK4 antagonize each other in their cytotoxicity towards sarcoma cells. CDK4 inhibition attenuates the induction of p53-responsive genes upon MDM2 inhibition. Moreover, the p53 response was also attenuated when co-depleting MDM2 and CDK4 with siRNA, compared to MDM2 single knockdown. The complexes of p53 and MDM2, as well as CDK4 and Cyclin D1, physically associated with each other, suggesting direct regulation of p53 by CDK4. Interestingly, CDK4 inhibition did not reduce p53 binding or histone acetylation at promoters, but rather attenuated the subsequent recruitment of RNA Polymerase II. Taken together, our results suggest that caution must be used when considering combined CDK4 and MDM2 inhibition for patient treatment. Moreover, they uncover a hitherto unknown role for CDK4 and Cyclin D1 in sustaining p53 activity.

## Introduction

Cyclin-dependent kinase 4 (CDK4) is a key promoter of cell proliferation. It enables the transition through the G1 phase of the cell cycle, a prerequisite for subsequent entry to S phase and cell division. Tumor cells often activate CDK4 to ensure proliferation, either by silencing genes that encode CDK4 antagonists or by enhancing CDK4 expression, e.g., through gene amplification. Pharmacological inhibitors of CDK4 have proven to be effective in cancer treatment, leading to the Food and Drug Administration (FDA) approval of Palbociclib (PD0332991), Ribociclib (LEE011) and Abemaciclib (LY2835319)^1^.

The MDM2 oncoprotein has also been extensively evaluated as a drug target. MDM2 antagonizes the tumor suppressor p53 by physical interaction and subsequent ubiquitination of p53. The interaction of p53 and MDM2 is amenable to targeting by small compounds, with Nutlin-3a (referred to here as “Nutlin”) representing the prototype^2^ and many similar and further refined compounds being developed ever since^3,4^. Preclinical analyses of MDM2 inhibitors have raised high expectations, especially when treating sarcoma^5^ or glioblastoma^6^ which contain *MDM2* gene amplifications. Clinical studies using MDM2-targeting drugs^7^, however, have currently not met the initial expectations^8^, at least not when used as single drugs. This has spurred the search for optimized combinations of MDM2 inhibitors with other cancer drugs.

Certain sarcomas, specifically liposarcomas, represent particularly promising cancer entities for treatment with MDM2 antagonists. These tumors contain amplifications of the *MDM2* gene in more than 90% of all cases^9^, and liposarcoma-derived cell lines undergo apoptosis when treated with MDM2-antagonizing drugs^5,10^. As expected, the response requires a wild-type p53 status and MDM2 overexpression^11^. However, attempts to treat liposarcoma patients with MDM2 antagonists have so far not yielded the expected clinical success^12,13^.

The amplification of the *MDM2* gene in sarcomas is often associated with *CDK4* amplifications^9^. Other examples of tumors containing both amplifications include melanomas^14^ and parosteal osteosarcomas^15^. Both genes are located close to each other on chromosome 12q13-15 but, nonetheless, the amplifications appear independent in most cases^9,16^. The co-amplification of both genes might constitute tumor cell addiction to the simultaneous activity of both gene products. This argued that targeting both MDM2 and CDK4 should yield synergistic tumor cell killing. And indeed, a recent report argued that this synergism might be achievable^17^. Moreover, a combination of MDM2 and CDK4 inhibitors is currently being evaluated in a Phase I clinical study (NCT02343172). However, in the previous report^17^, the impact of drug combinations on tumor growth was only marginally increased when compared to CDK4 inhibitor alone. Thus, potential synergies between CDK4 and MDM2 inhibitors remain to be investigated.

Here we show that the inhibition of CDK4 attenuates MDM2 inhibitor-induced activity of p53, leading to decreased rather than synergistic cytotoxicity. In parallel, the complexes of MDM2 and p53, as well as CDK4 and Cyclin D1, physically associate with each other. CDK4 inhibition still allows efficient binding of p53 to its target genes. In contrast, combined inhibition of CDK4/6 and MDM2 led to diminished RNA Polymerase II recruitment and thus decreased transcription of p53 target genes.

## Materials and methods

### Cell culture, treatment, siRNA and plasmid transfections

Human CRL-3043 (93T449) and CRL-3044 (94T778) cell lines were purchased from ATCC. GOT-3 cells were a gift from Pierre Åman, University of Gothenburg, Sweden. SJSA and H1299 cells were obtained from the German Collection of Cell lines (DSMZ, Braunschweig) and maintained in Dulbecco’s modified Eagle’s medium. CRL-3043, CRL-3044 and GOT-3 cells were maintained in RPMI medium. Cell culture media were supplemented with 10% fetal bovine serum and antibiotics including penicillin/streptomycin and ciprofloxacin. Cells were maintained at 37 °C in a humidified atmosphere with 5% CO_2_. For treatment of cells, neocarzinostatin (NCS, 0.5 mg/mL, Sigma-Aldrich), Nutlin-3a (Sigma N6287 and BOC life sciences 675576-98-4), Palbociclib (PD0332991 isethionate, Sigma PZ0199), Ribociclib (LEE011, Selleckchem S7440), Abemaciclib (LY2835219, Selleckchem S7158) and MG-132 (Calbiochem 474791) were diluted in pre-warmed medium and added to the cells for the indicated periods of time. For small interfering RNA (siRNA)-mediated transfection using Lipofectamine 2000 (Life Technologies), cells were reverse transfected with 10 nM siRNA to MDM2 (Ambion; custom made, AAGCCAUUGCUUUUGAAGUUAtt (sense), UAACUUCAAAAGCAAUGGCUUtt (antisense)); CDK4 (s2822, Ambion) and a negative control siRNA. Medium was changed after 24 h and cells were harvested 24 h later. For plasmid overexpression, 2 µg of plasmid was transfected along with Lipofectamine 2000 by forward transfection. Medium was changed after 4 h and the cells were harvested 24 h later.

**Table:** 

Plasmid	Origin
pCMV6XL5	Origene
pCMV-MDM2	B. Vogelstein^18^
pCMV-cyclin D1	Addgene 19927

### Quantitative mRNA analysis by qRT-PCR

Total RNA was isolated using TRIzol (Invitrogen), followed by reverse transcription with Moloney Murine Leukemia Virus reverse transcriptase and random hexamer primers (Thermo Scientific). Maxima SYBR Green master mix (Invitrogen) was used for real-time PCR. Gene expression levels were normalized to the messenger RNA (mRNA) from the *RPLP0* gene and the analysis was conducted using the ΔΔCt method. Quantitative real-time polymerase chain reaction (qRT-PCR) primer sets were chosen as follows:

**Table:** 

RPLP0 for	GATTGGCTACCCAACTGTTG
RPLP0 rev	CAGGGGCAGCAGCCACAAA
p21/CDKN1A for	TAGGCGGTTGAATGAGAGG
p21/CDKN1A rev	AAGTGGGGAGGAGGAAGTAG
MDM2 for	TCAGGATTCAGTTTCAGATCAG
MDM2 rev	CATTTCCAATAGTCAGCTAAGG
PUMA/BBC3 for	GCCAGATTTGTGAGACAAGAGG
PUMA/BBC3 rev	CAGGCACCTAATTGGGCTC
PIG3/TP53I3 for	GCTTCAAATGGCAGAAAAGC
PIG3/TP53I3 rev	GTTCTTGTTGGCCTCCATGT
p21/CDKN1A i.e. for	GTGGCTATTTTGTCCTTGGGC
p21/CDKN1A i.e. rev	TGGCAGATCACATACCCTGTTC
MDM2 i.e. for–a	CGGAGAGTGGAATGATCCCC
MDM2 i.e. rev–a	GCTGGGAACCAGCGATAGAG
MDM2 i.e. for–b	CCACAGATGTTTCATGATTTCCAG
MDM2 i.e. rev–b	AGGTGGTTACAGCACCATCAG
MDM2 i.e. for–c	AGGAGATTTGTTTGGCGTGC
MDM2 i.e. rev–c	GGTGAACTGAAATGTTAGCCCAG

### Immunoblot analysis

As previously described^19^, cells were harvested in protein lysis buffer (1% Triton-X 100, 1% sodium deoxycholate, 0.1% SDS,150 mM NaCl, 10 mM EDTA, 20 mM Tris-Hcl pH 7.5, 2 M urea). After scraping the cells on ice with the lysis buffer, samples were briefly sonicated for 10 min at high speed to disrupt DNA-protein complexes. The total amount of protein present in each sample was measured using Pierce BCA Protein assay kit (Thermo Scientific Fisher). Prior to loading the samples on sodium dodecyl sulfate–polyacrylamide gel electrophoresis (SDS-PAGE), they were denatured in 6× Laemmli buffer at 95 °C for 5 min. Equal amounts of protein samples were separated by SDS-PAGE. This was followed by transfer on a nitrocellulose membrane and visualization with the following antibodies: pH2AX (S139) (9718, Cell Signalling), Beta-Actin (ab8227, Abcam), p21 (2947, Cell Signalling), pRb (S807/811) (9308, Cell Signalling), Rb (9309, Cell Signalling), MDM2 (OP 46, Calbiochem), p53 (DO-1, sc-126, Santa Cruz), p53-HRP (DO-1, sc-126, Santa Cruz), CDK4 (ab68266 abcam; DCS-35, sc-23896, Santa Cruz), p53 K382ac (2525, Cell Signalling) and Cyclin D1 (ab134175, Abcam).

### Cell proliferation assay (Celigo)

To determine the proliferation of cells under different treatment conditions, cells were seeded at a density of 6 × 10^3^ cells/well in 24-well plates. They were treated with Palbociclib and/or Nutlin, with dimethyl sulfoxide (DMSO) as the control, at the indicated concentrations. Their proliferation capacity was measured using the Celigo Cytometer (Nexcelom, software version 2.0). Cell confluence in triplicate samples was measured every 24 h for up to 8 days.

### Cell viability assay

In order to measure cell viability after drug treatment, the cells were seeded at a density of 2 × 10^4^ cells/well in 96-well plates with white walls and bottom. These cells were treated with Nutlin and/or Palbociclib at the indicated concentrations with the highest concentration of DMSO as a control. The drugs were incubated for 48 or 72 h as indicated. Following this, luminescence was measured using the CellTiter-Glo® Luminescence Cell Viability Assay (Promega). The CellTiter-Glo® Reagents were mixed and added in a 1:1 ratio to each well. The solutions were incubated in an orbital shaker for 10 min to facilitate lysis of the cells. Subsequently, the luciferase signal was measured on a LuminometerDLReady™Centro LB 960 reader and the measurements for each condition were processed.

### Cell cycle analysis

To analyze the cell cycle profile under different treatment conditions, the cells were trypsinized and centrifuged to obtain pellets. To each cell pellet, 100% ethanol was added for fixation overnight. Following fixation, the cells were washed to allow for rehydration. Finally, for cell cycle analysis, propidium iodide was added and the profiles were obtained using the Guava flow cytometry system (Millipore). Three biological replicates were processed for each condition using the same gate settings.

### Protein co-immunoprecipitation

To carry out endogenous co-immunoprecipitation (Co-IP) in SJSA cells, individual 15 cm dishes were used for precipitation with one antibody. The cells were harvested in Co-IP buffer (50 mM Tris-HCl pH 7.5, 300 mM NaCl, 1% NP-40 and 0.1% sodium deoxycholate) along with protease inhibitors (Roche). The homogenized cell lysates were pre-cleared with Protein G Sepharose beads (GE Healthcare). Equal amounts of cell lysates were used for overnight precipitation along with 3 μg of each antibody. The following day, the cell lysates were incubated with Protein G Sepharose beads for 2 h. Subsequently, the samples were washed using the Co-IP buffer and the beads were re-suspended in 6× Laemmli Buffer. These samples were subjected to SDS-PAGE followed by immunoblot analysis. For exogenous Co-IP, cells were transfected in 6-well plates with plasmids 24 h prior to harvesting. The IP procedure was done as described above, using one well per antibody precipitation.

### Chromatin immunoprecipitation

Chromatin immunoprecipitation (ChIP) was done according to the protocol published by Denissov and colleagues^20,21^. Cells were fixed in 1.1% formaldehyde for 30 min, quenched with 0.125 M glycine and lysed in a buffer containing 0.1% SDS, 1% Triton-X 100, 0.15 M NaCl, 1 mM EDTA and protease inhibitors. Sonication was done using the Bioruptor Pico sonication device (Diagenode) in Bioruptor Microtubes for 15 cycles. The samples were subjected to incubation with antibody (2 μg) and Protein A/G PLUS-Agarose (Santa Cruz) beads overnight. Bead interaction was released after 20 min of rotation incubation in 1% SDS and 0.1 M NaHCO_3_, and the DNA-protein crosslink was reversed by the addition of 0.2 M NaCl and shaking at 65 °C for 4–5 h. DNA was purified using the MiniElute PCR Purification Kit (Qiagen) and used for targeted PCR. For IP, the following antibodies were used: p53 (DO-1, sc-126, Santa Cruz), IgG (ab46540, Abcam), H3K27ac (C15410196, Diagenode), RNA Polymerase II (MABI0601, MBL Life Sciences; sc-17798, Santa Cruz; sc-899, Santa Cruz). The following primers were used for targeted ChIP:

For transcription start site amplification

**Table:** 

p21/CDKN1A for	CTTTCTGGCCGTCAGGAACA
p21/CDKN1A rev	CTTCTATGCCAGAGCTCAACATGT
MDM2 for	TTCAGTGGGCAGGTTGACTC
MDM2 rev	CCAGCTGGAGACAAGTCAGG
Puma/BBC3 for	CCCTGCTCTGGTTTGGTGAG
Puma/BBC3 rev	AGTCACTCTGGTGAGGCGAT
PIG3/TP53I3 for	CCCTGGGTACCTGCATTAAG
PIG3/TP53I3 rev	TAGCCGTGCACTTTGACAAG
myo for	CTCATGATGCCCCTTCTTCT
myo rev	GAAGGCGTCTGAGGACTTAAA
p21/CDKN1A TR for	CCAGGGCCTTCCTTGTATCTCT
p21/CDKN1A TR rev	ACATCCCCAGCCGGTTCT
TFF1 6 kb for	CAGGCTTCTCCCTTGATGAAT
TFF1 6 kb rev	ACACCCACCTTCCACAACAC
HNRNPK for	ATCCGCCCCTGAACGCCCAT
HNRNPK rev	ACATACCGCTCGGGGCCACT

For H3K27ac ChIP

**Table:** 

p21 TSS(K27ac) for	TCAGGTGAGGAAGGGGATGG
p21 TSS(K27ac) rev	TGTCGCAAGGATCTGCTGG
MDM2 TSS(K27ac) for	AGATGGAGCAAGAAGCCGAG
MDM2 TSS(K27ac) rev	GTACGCACTAATCCGGGGAG
p21_u2.2kb_for	AGCAGGCTGTGGCTCTGATT
p21_u2.2kb_rev	CAAAATAGCCACCAGCCTCTTCT

### RNA sequencing

RNA sequencing was carried out as previously described^19^. Briefly, the quality of total RNA was determined using the Bioanalyzer 2100 from Agilent Genomics. All samples analyzed exhibited an RNA Integrity Number of >8. To prepare libraries from 1 µg of total RNA, the TruSeq RNA LT Sample PrepKit (Illumina) was employed. Barcodes for sample preparation were used according to the manufacturer’s instructions. Accurate quantitation of complementary DNA (cDNA) libraries was performed with the QuantiFluor™dsDNA System (Promega). The size range of final cDNA libraries was determined applying the DNA 1000 chip on the Bioanalyzer 2100. cDNA libraries were amplified and sequenced via cBot and HiSeq 4000 (Illumina; SR, 1 × 50 bp, 6 Gb/sample ca. 40–50 million reads per sample). Sequence images were transformed using the Illumina software BaseCaller to bcl files, which were demultiplexed to fastq files with CASAVA (version 1.8.2). Quality check was performed via FastQC (version 0.10.1 Babraham Bioinformatics). Fastq files were mapped to the human reference transcriptome (UCSC hg19) using TopHat gapped-read mapper with very sensitive Bowtie 2 settings on Galaxy Platform (Version 0.9), Bowtie 2 (version 2.1.)^22^. The read counting was performed via HTSeq^23^ (version 0.6.0) with the following parameters: -f bam -r pos -s reverse -a 10 -t exon -m union. The count files were subsequently subjected for differential analysis using the DESeq2 package^24^ on R (Bioconductor version 3.2.2). Heatmap was generated using *z*-score analysis. RNA library preparation and sequencing was done by the Transcriptome Analysis Laboratory (TAL, Göttingen).

### Statistical testing

Statistical testing was performed using Graph Pad Prism 6. An unpaired *t*-test was calculated and multiple comparisons were corrected using the Sidak Bonferroni method with an assumed significance for *p*- values ≤ 5%. Asterisks represent significance in the following way: ***p ≤ 0.001; **p ≤ 0.01; *p ≤ 0.05.

## Results

### Inhibitors of CDK4 and MDM2 lack synergistic cytotoxicity towards sarcoma cells

**Fig. 1:**
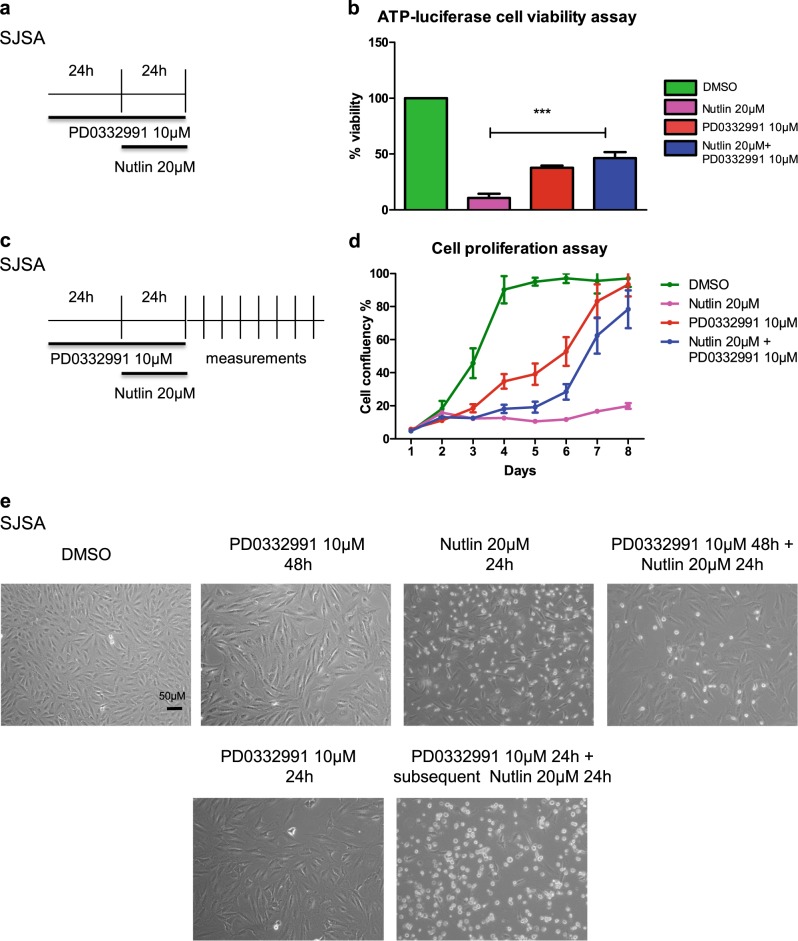
CDK4 inhibitors and MDM2 antagonists fail to synergize with regard to cytotoxicity towards sarcoma cells. **a** SJSA cells were treated with DMSO, Nutlin, PD0332991 and its combination at the indicated concentrations adhering to the depicted schedule. **b** Cell viability was measured by quantification of the ATP content. The combination of PD0332991 and Nutlin showed a protective effect in comparison to Nutlin alone. Mean of two biological replicates. *** denotes p ≤ 0.001. **c** Schedule to determine cell proliferation upon drug treatment. **d** Cell proliferation was assessed by daily measuring the confluency of cells using a Celigo cell cytometer. The medium was changed every 24 h. Nutlin treatment reduced cellular proliferation. However, pretreatment with PD0332991 in combination with Nutlin led to increased cell numbers, indicating that the two drugs do not synergize but rather act in an antagonistic fashion. Mean of three biological replicates. **e** Morphology of SJSA cells, observed by bright field microscopy. Upon treatment with Nutlin, the cells shrank and detached. Pretreatment with PD0332991 protected the cells from this cytotoxic effect of Nutlin. However, removal of PD0332991 followed by Nutlin treatment did not result in cell protection. The scale bar (50 μm) applies to all images in this panel

Given the co-amplification of the *MDM2* and *CDK4* genes in sarcoma, we sought to test whether the combined inhibition of both gene products might synergistically eliminate cancer cells. We treated SJSA cells (osteosarcoma cells with amplifications of MDM2 and CKD4, cf. www.cbioportal.org and Fig. S1a) with combinations of the CDK4 inhibitor Palbociclib (PD0332991) and the MDM2 antagonist Nutlin. As expected, Nutlin induced p53 accumulation and its target genes p21 and MDM2 (Fig. S1b), and PD0332991 abolished the phosphorylation of the CDK4 substrate pRb at Serine 807/811^25^ (Fig. S1c). Nutlin also reduced pRb phosphorylation, likely due to the induction of the CDK4 inhibitor p21. Importantly, Nutlin profoundly decreased the viability of SJSA cells, as reported previously^26^, and Palbociclib also reduced their viability (Fig. 1a, b). Strikingly, however, Palbociclib completely failed to enhance the cytotoxic effects elicited by Nutlin. Instead, Nutlin-treated cells even survived to a significantly greater extent when co-treated with Palbociclib. Similarly, the long-term survival of SJSA cells was strongly decreased by Nutlin alone, but was rescued by co-treatment with Palbociclib (Fig. 1c, d). Moreover, SJSA cells treated with Nutlin alone displayed morphology with round and shrunk cells, corresponding to apoptosis^27^ (Fig. 1e). Again, this occurred to a lesser extent when the cells had first been treated with Palbociclib. Combination of the drugs at different concentrations in various *MDM2*-amplified sarcoma cell lines also revealed no synergism with regard to viability of cells (Fig. S1d–g; note that here viability was assayed immediately after 72 h of treatment, without allowing the cells to further proliferate). Taken together, these results strongly suggest that, at least under some circumstances, Palbociclib is capable of antagonizing the cytotoxic activity of Nutlin.

### CDK4 is required for p53-induced gene expression

**Fig. 2:**
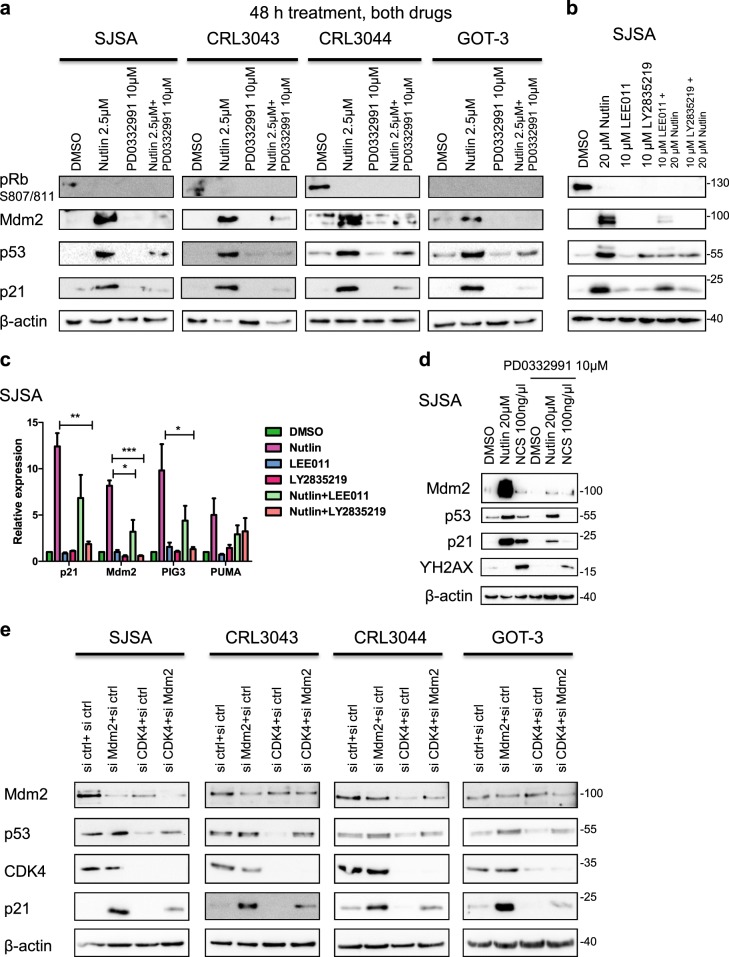
CDK4 is required for p53-induced gene expression. **a** SJSA (osteosarcoma), CRL-3043, CRL-3044 and GOT-3 (all liposarcoma) cells were treated with DMSO, Nutlin, PD0332991 or the drug combination for 48 h. Cells were harvested for immunoblot analysis to detect p53 and its target gene products p21 and MDM2. Upon Nutlin treatment, increased levels of MDM2, p53 and p21 were observed. However, the combination of Nutlin with PD0332991 decreased p53-induced target gene expression. pRb phosphorylated at 807/811 was detected as a positive control for the activities of Nutlin and PD0332991. β-Actin served as loading control. **b** SJSA cells were treated with the alternate CDK4/6 inhibitors Ribociclib (LEE011) and Abemaciclib (LY2835219) for 30 h, alone or in combination with Nutlin at 20 µM for 6 h. Immunoblot analysis was performed as in (**a**). Like Palbociclib (PD0332991), the combination of Nutlin with alternate CDK4/6 inhibitors diminished p53 target gene expression when compared to Nutlin treatment alone. **c** SJSA cells were treated with the alternate CDK4/6 inhibitors Ribociclib (LEE011) and Abemaciclib (LY2835219), alone and in combination with Nutlin as in (**b**). mRNA levels corresponding to p53 target gene expression were assessed by quantitative real-time PCR. RPLP0 was used as a reference gene. Again, p53-induced gene expression was found diminished by CDK4/6 inhibitors. Mean of three biological replicate. ***p ≤ 0.001; **p ≤ 0.01; *p ≤ 0.05. **d** SJSA cells were treated with DMSO, Nutlin, Neocarzinostatin (NCS), PD0332991 and their combinations at the indicated concentrations for 6 h. Lysates were subjected to immunoblot analysis. Upon NCS treatment, increased levels of p21 and ϒ-H2AX were found, indicative of a DNA damage response. As in the case of Nutlin, NCS-induced p53 activity, revealed by p21 accumulation, was found reduced by the CDK4/6 inhibitor. **e** SJSA, CRL-3043, CRL-3044 and GOT-3 cells were depleted of endogenous MDM2, CDK4 by siRNA transfection, in comparison to control (ctrl) siRNA. Cells were harvested for immunoblot after 48 h to detect p53 and its target gene product p21. Upon depletion of MDM2, the expected increase in p21 and p53 levels was observed. In contrast, the co-depletion of CDK4 along with MDM2 induced p21 and p53 levels only to a lesser extent

To further investigate why CDK4 inhibition attenuates Nutlin-induced cell death, we asked whether CDK4 inhibitors might interfere with p53-induced gene expression. A panel of sarcoma cells, including SJSA cells, as well as CRL-3043^28^, CRL-3044^28^ and GOT-3^29^ (the latter three derived from well-differentiated liposarcoma and with amplifications of CDK4 and MDM2 genes), were treated with Palbociclib to inhibit CDK4, as well as Nutlin to block the MDM2–p53 interaction. The levels of p53-responsive gene products were then assessed by immunoblot analysis. Nutlin alone increased the levels of p53 and its target gene products MDM2 and CDKN1A/p21, as expected. In contrast, Palbociclib alone had little effect on them, while it did reduce the amount of phosphorylated pRb. Importantly, however, in combination with Nutlin, Palbociclib markedly decreased the protein levels of the p53 target gene products MDM2 and p21 (Fig. 2a). To determine whether the impairment of p53 target gene expression was specific to Palbociclib or whether it was due to inhibition of CDK4/6 kinase activity, in general, we treated SJSA cells with alternate, FDA-approved CDK4/6 inhibitors, namely LEE011 (Ribociclib) and LY2835219 (Abemaciclib), alone or in combination with Nutlin. Again, p53 target gene expression was decreased when Nutlin was combined with the CDK4/6 inhibitors at the protein (Fig. 2b) and mRNA (Fig. 2c) levels. Thus, CDK4 inhibition generally reduces p53 activity. The failure of Nutlin to fully induce pro-apoptotic genes in the presence of CDK4 inhibitor may also explain why CDK4 inhibition attenuates the cytotoxic effects of Nutlin (Fig. 1). We next increased p53 activity by inducing a DNA damage response, which enhances p53 phosphorylation through the kinases ATM and Chk2^30^. To this end we used NCS, a radiomimetic compound that induces double-strand DNA breaks in a manner similar to ionizing radiation^31^. Strikingly, Palbociclib pretreatment strongly decreased the accumulation of p53 and its target gene product p21 in response to NCS (Fig. 2d). Hence, CDK4 inhibition can also interfere with the p53-inducing ability of DNA damaging drugs, giving rise to caution when combining CDK4 inhibitors with conventional chemotherapy in cancer treatment. Furthermore, we performed analogous investigations replacing pharmacological inhibitors with siRNAs targeting MDM2 and CDK4. This revealed corresponding changes in p53-induced p21, i.e., induction by MDM2 knockdown alone, but far less induction by the simultaneous depletion of MDM2 and CDK4 (Fig. 2e). Similarly, impaired p53 target gene expression upon CDK4 inhibition was also observed when using different time schedules, drug concentrations or the alternate MDM2 antagonist RG-7388 (Fig. S2, a–e). Moreover, to exclude any role of the p53 kinase HIPK2^32,33^ in this context, we performed a parallel experiment replacing CDK4 inhibitors with the HIPK2 inhibitor A64 (PubChem Substance ID 329826044) but did not observe any detectable change in p53 activity (Fig. S2, f, g). Taken together, these results strongly suggest that CDK4 inhibition or depletion severely diminishes the transcriptional activity of p53 in response to MDM2 antagonists. This raises an important potential caveat regarding the combination of CDK4 inhibitors with p53-activating drugs for cancer therapy.

### Short-term reactivation of CDK4 is sufficient to rescue p53 activity

**Fig. 3:**
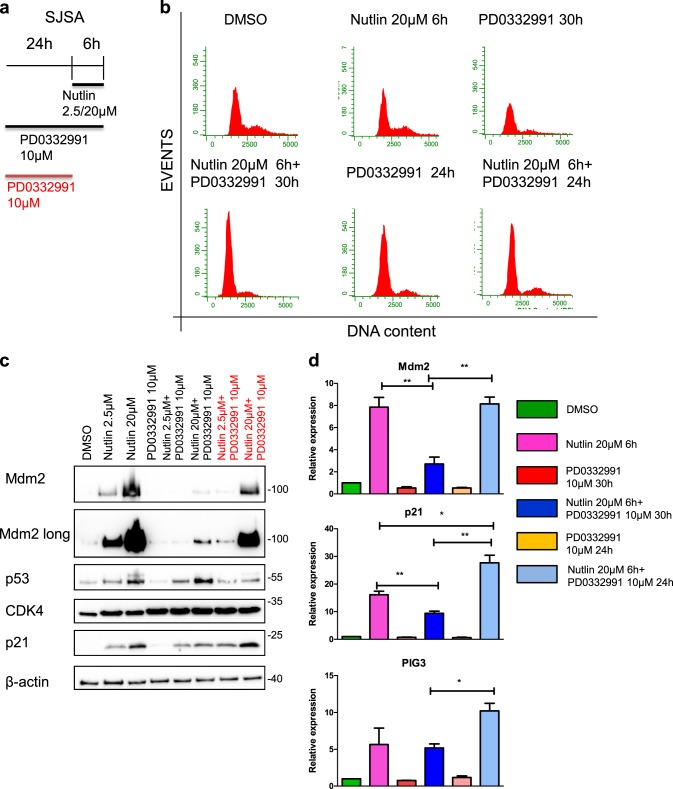
Short-term reactivation of CDK4 is sufficient to rescue p53 activity which is independent of cell cycle. **a** SJSA cells were treated as indicated in the schedule. **b** Flow cytometry analysis of the DNA content. PD0332991 induced cell cycle arrest in G1, irrespective of the (short-term) Nutlin treatment. Representative images of two biological replicate. **c** To investigate the p53 target gene expression, immunoblot analysis was carried out. Nutlin treatment led to the accumulation of p53, p21 and MDM2, which was diminished by PD0332991. Upon removal of PD0332991 during Nutlin treatment, however, p21 and MDM2 levels were restored. **d** Cells were treated as in (**a**–**c**), followed by quantitative RT-PCR to quantify the expression of the p53 target genes MDM2, p21 and PIG3, in comparison to the reference gene RPLP0. Nutlin induced these genes while PD0332991 significantly decreased their expression levels. Removal of PD0332991 during Nutlin addition reactivated p53 target gene expression. Mean of three biological replicates. **p ≤ 0.01; *p ≤ 0.05

To test whether the impact of CDK4 inhibition on p53 activity is mediated by cell cycle arrest, we analyzed the timing needed for the two drugs to interact. One possibility for the observed effects is that a cell cycle arrest induced by CDK4 inhibition might impair p53 activity. As expected, flow cytometric analyses verified that Palbociclib arrested the majority of treated cells in G1 (Fig. 3a, b). Although cell cycle arrest is not generally considered as a way to inhibit p53 activity, we still investigated whether this arrest in G1 represents the reason for the attenuated p53 response. To this end, we removed the CDK4/6 inhibitor from the cells for 6 h (compared to continued CDK4 inhibition) and then immediately treated with Nutlin. This brief removal of the CDK4 inhibitor was not enough to resume the cell cycle, at least as far as can be judged by propidium iodide staining (Fig. 3b). However, p53 activity was still markedly increased upon Palbociclib removal, when compared to cells that were treated with Nutlin in the continued presence of Palbociclib, as determined by immunoblot analysis of p53 target gene products (Fig. 3c) and also by assessing the mRNA levels corresponding to such genes (Fig. 3d). Thus, we conclude that CDK4 activity is required for maximal p53-induced gene expression, regardless of cell cycle progression.

### CDK4 inhibition attenuates the expression of a broad range of p53-induced genes

**Fig. 4:**
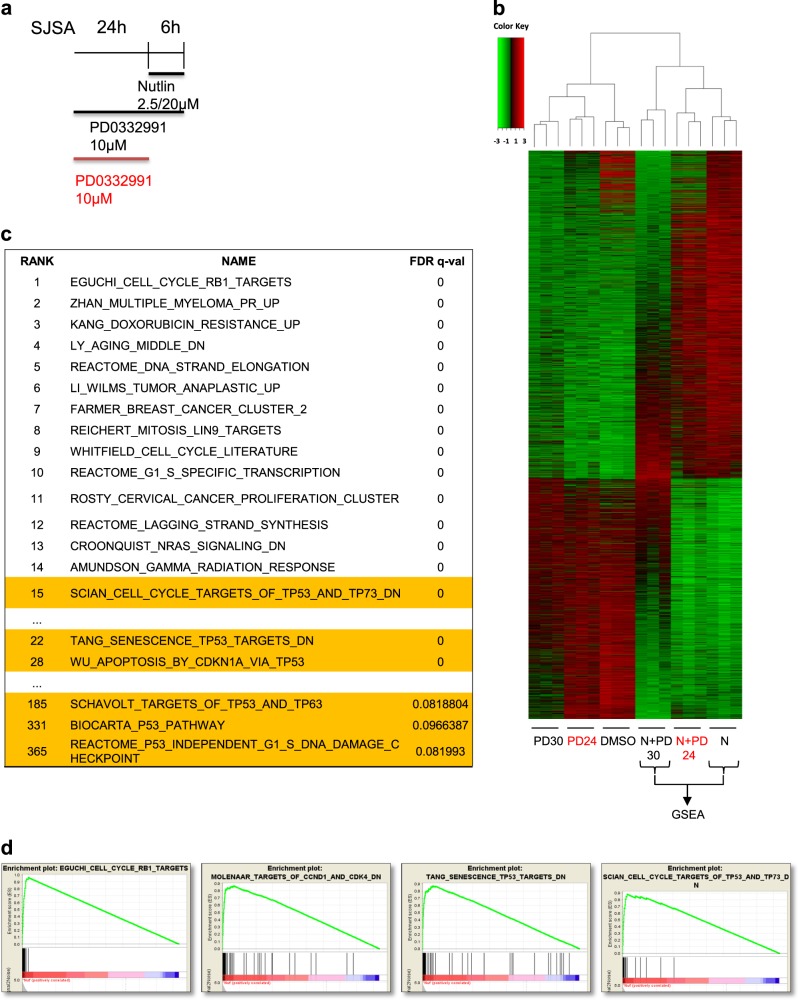
CDK4 inhibition attenuates the expression of a broad range of p53-responsive genes. **a** SJSA cells were treated with DMSO, Nutlin, PD0332991 and its combination as indicated in the schedule, followed by RNA deep sequencing analysis. **b** Heatmap depicting differentially regulated genes sorted according to the *z*-scores after performing DeSEQ for the condition of Nutlin vs DMSO (*n* = 2368). Only genes with base mean >20, log2 fold >1, log2 fold <−1 and adjusted *p* value < 0.05 were taken into consideration. Six samples with biological triplicate are represented. N refers to Nutlin, PD refers to PD0332991 and DMSO as control. RNA-sequencing data of this study were submitted to the GEO, GSE113369. **c** Gene Set Enrichment Analysis (GSEA) from C2 curated gene sets, provided by the Molecular Signatures Database (MSigDB) v5.0^52^, was performed using variance stabilized RNA-Seq reads from Nutlin (represented as N) and Nutlin+PD0332991 (represented as N+PD30) treated samples. The table was generated by selecting false discovery rate (FDR) < 25% and Enrichment Score (ES) in the descending order. The complete list of pathways is provided in Supplementary Table 2. **d** Selected enrichment plots from gene sets induced by Nutlin vs Nutlin+PD0332991 are provided as examples

Next, we assessed the extent to which the induction of genes by p53 is affected by CDK4 inhibition, and whether CDK4 inhibition might display a similarly broad impact on unrelated gene sets. We treated SJSA cells with Palbociclib and/or Nutlin (Fig. 4a), followed by next-generation RNA sequencing analysis (RNA-Seq). This approach revealed that most p53-responsive genes, as identified through their induction by Nutlin, were expressed to a lesser degree when cells were pretreated with the CDK4/6 inhibitor (Fig. 4b). Comparing Nutlin-treated cells with or without CDK4 inhibitor, it was still the p53-responsive genes that were differentially expressed to the greatest extent after the cell cycle targets, as determined by gene set enrichment analysis (GSEA) (Fig. 4c, d). We conclude that, when cells are subjected to MDM2 inhibition, a CDK4/6 inhibitor specifically attenuates the expression of p53-responsive genes more than any other distinguishable group of genes except the cell cycle regulators. This suggests that CDK4 activity has a direct and specific impact on p53-induced transcription.

### p53 physically interacts with CDK4 and Cyclin D1

**Fig. 5:**
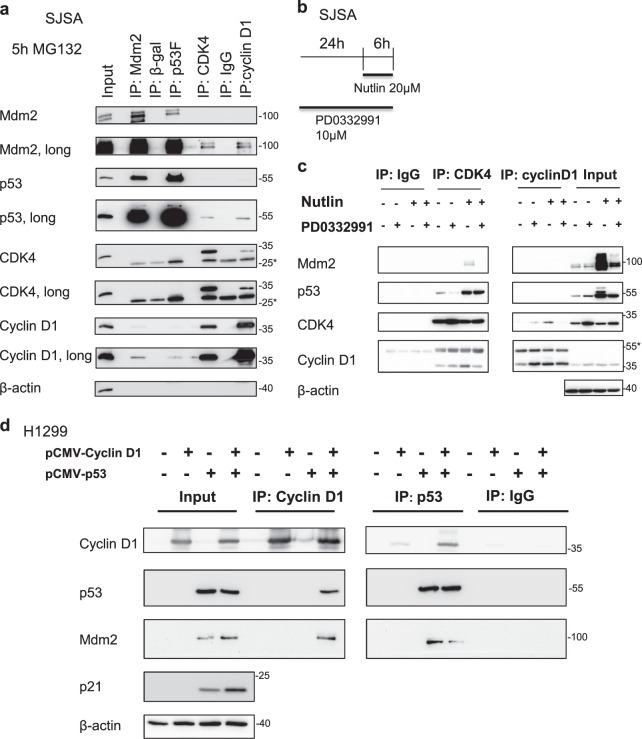
Association of the CDK4/Cyclin D1 complex and the p53/MDM2 complex. **a** Co-immunoprecipitation of endogenous proteins was carried out with lysates of SJSA cells treated with the proteasome inhibitor 20 µM MG-132 for 5 h. Antibodies to precipitate MDM2 or p53 were compared with an anti-beta-galactosidase antibody as a control, and antibodies to CDK4 and Cyclin D1 were compared to pre-immune IgG. Immunoblot analysis of the precipitated material showed association of the CDK4/Cyclin D1 complex with the MDM2/p53 complex. Representative figure of three biological repeats. *Indicates the immunoglobulin heavy chain IgL. **b** Scheme used for the treatment of SJSA cells with Nutlin and PD033299. **c** Co-immunoprecipitation was performed from lysates of SJSA cells as described in (**b**). Upon Immunoprecipitation (IP) with antibodies to CDK4, p53 accumulation was observed. When directly precipitating CDK4 or Cyclin D1 complex formation between the two decreased upon combined treatment, in comparison to single treatment with Nutlin alone (quantification of the bands in Fig. S3). *Indicates the immunoglobulin heavy chain IgH. **d** Co-immunoprecipitation was carried out from lysates of H1299 cells upon plasmid-based overexpression of p53 or Cyclin D1, revealing the association of the two, and also the association of MDM2 with p53 in this context

To elucidate how CDK4 might affect the activity of p53, we tested whether the two molecules might physically associate with each other. We tested this in cell lysates from SJSA cells treated with a proteasome inhibitor. Indeed, co-immunoprecipitation analyses revealed the interaction of the MDM2–p53 and the CDK4–Cyclin D complexes (Fig. 5a). The interaction of p53 and CDK4 was also found in Nutlin-treated cells (Fig. 5b, c). Combined inhibition of MDM2 and CDK4 led to decreased association of Cyclin D1 and CDK4 when compared to Nutlin alone (Fig. 5c with quantification in Fig. S3). Possibly, the CDK4 inhibitor diminishes the proper folding of CDK4^34^ and thus reduces Cyclin D1 binding. The interaction of p53 with Cyclin D1 was further confirmed by plasmid-based overexpression of both components (Fig. 5d). In sum, p53 physically associates with CDK4 and Cyclin D1, suggesting a mechanism by which CDK4 might directly regulate the activity of p53 as a transcription factor.

### CDK4 inhibition does not interfere with p53 binding to its cognate promoter elements but diminishes the recruitment of RNA Polymerase II

**Fig. 6:**
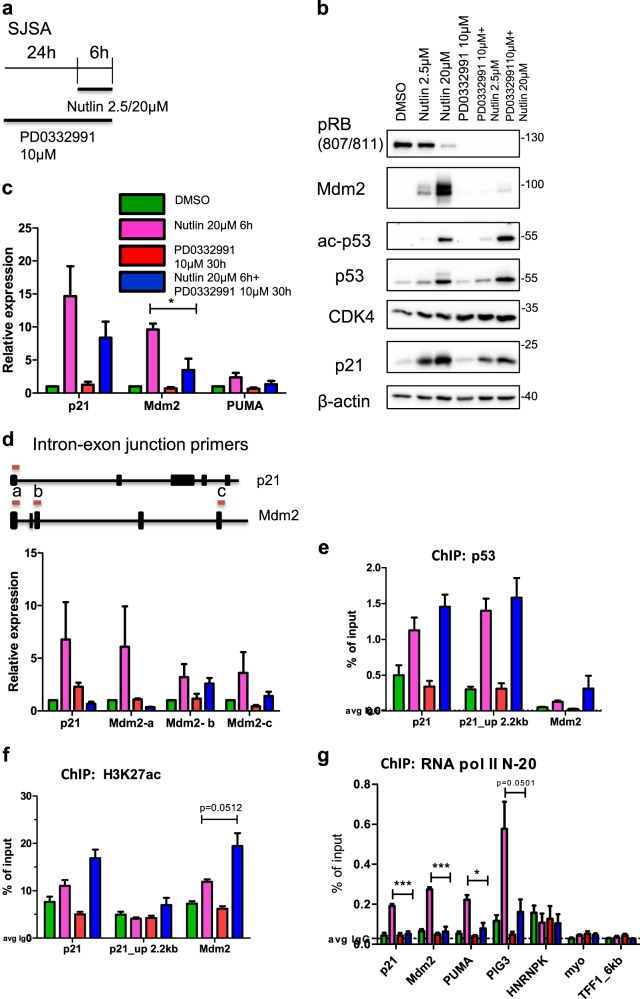
CDK4 inhibition does not interfere with p53 binding to its cognate promoter elements. **a** Scheme indicating the treatment regimen used for the individual and combinatorial treatment of SJSA cells with Nutlin and/or PD0332991. **b** Immunoblot analysis following treatment as in (**a**). Apart from p53 and its target gene products p21 and MDM2, the acetylation of p53 at Lys 382 was detected. This acetylation was increased, rather than attenuated, upon the combined treatment of Nutlin with PD0332991 in comparison to Nutlin treatment alone. **c** Quantitative real-time PCR was carried out to quantify mRNA expression levels of p53 target genes upon treatment as in (**a**), with similar results as in Fig. 2c. Mean of four independent experiments. ***p ≤ 0.001; **p ≤ 0.01; *p ≤ 0.05. **d** Primers spanning intron–exon junctions were used to quantify pre-mRNA for p21 and MDM2. The origin of the PCR products, with respect to the gene structures, are indicated in the figure. Upon treatment as in (**a**), SJSA cells were subjected to mRNA analysis. It was observed that nascent RNA of p21 and MDM2 was increased upon Nutlin treatment. With the combination, the relative gene expression was reduced, in particular at sites proximal to the promoter. Mean of two independent experiments. **e** Chromatin Immunoprecipitation of p53 was carried out upon treatment with Nutlin and PD0332991 as in (**a**). The occupancy of promoters by p53 remained similar with Nutlin treatment, compared with the combination of Nutlin with PD0332991, at the transcriptional start site (TSS) of p53 target genes p21 and MDM2, and at an enhancer site on p21 which contains another p53-responsive element. IgG was used as a negative control. Mean of four independent experiments. **f** Upon chromatin immunoprecipitation of histone H3 with acetylation at K27, followed by quantitative real-time PCR at the TSSs of p21 and MDM2 as well as the enhancer site of p21, we observed that the enrichment of H3K27ac was increased upon the combination of Nutlin and PD0332991 when compared to Nutlin treatment alone. IgG is used as a negative control. Mean of three biological replicate. **g** Immunoprecipitation for the enzyme pivotal for transcription, RNA Polymerase II. We observed that at the TSSs of the p53 target genes p21/CDKN1A, MDM2, PUMA/BBC3, and PIG3/TP53I3, RNA Polymerase II was enriched with Nutlin treatment. Upon combining this with the CDK4/6 inhibitor, we observed decreased occupancy at the p53 TSS sites. HNRNPK, TFF1_6 kb (6 kb downstream region of TFF1 gene), and myo served as negative controls, not associating with the RNA Polymerase II. IgG is used as a negative control as well. Mean of three biological repeats. ***p ≤ 0.001; **p ≤ 0.01; *p ≤ 0.05

To mechanistically understand how CDK4/6 inhibition reduces the activity of p53 as a transcription factor, we performed immunoblot analysis to detect the acetylation of p53 on Lys 382, an activating modification of p53^35,36^. Surprisingly, we observed that the acetylation of p53 was even stronger when PD0332991 was combined with Nutlin than Nutlin alone (Fig. 6a, b). In contrast, we still observed decreased expression of p53 target genes, at the protein (Fig. 6b) and mRNA (Fig. 6c) level, when adding the CDK4/6 inhibitor to Nutlin. To determine differences in nascent pre-mRNA levels, we designed PCR primers spanning exon–intron boundaries of p53-responsive transcripts, namely p21 and MDM2. We observed that CDK4/6 inhibition mostly reduced pre-mRNA levels proximal to the promoters of p53-responsive genes in response to Nutlin (Fig. 6d), arguing that the initiation of transcription depends most strongly on cyclin-dependent kinases. Finally, we performed ChIP analyses to examine p53 occupancy on target gene promoters. Nutlin increased the amount of p53 associated with its cognate promoter elements, as expected. Interestingly, however, CDK4/6 inhibition did not decrease but even further increased the extent of p53 occupancy on target gene promoters (Fig. 6e), despite the decreased expression of the respective target genes. Similarly, the acetylation of histone H3 at lysine 27 (H3K27ac) at p53-responsive promoters was not impaired, but rather increased (Fig. 6f), still in line with the notion that p53 binding and the subsequent recruitment of histone acetyltransferases^35^ is still intact on these promoters. On the other hand, however, the association of RNA Polymerase II with p53-responsive genes was decreased in response to CDK4/6 inhibition (Fig. 6g), suggesting that CDK4/6 activity is required for p53-mediated recruitment of RNA Polymerase II. This was confirmed by two different antibodies to RNA Polymerase II (Fig. S4a–c). In conclusion, CDK4/6 inhibition interferes with the recruitment of RNA Polymerase II by p53, thereby diminishing the initiation of transcription at p53 target genes.

## Discussion

Despite the co-amplification of genes encoding CDK4 and MDM2 in human malignancies, our results indicate that targeting both simultaneously may be counterproductive for cancer therapy. CDK4 inhibition attenuates the p53 response to MDM2-targeted drugs, resulting in decreased cytotoxicity. The CDK4–Cyclin D1 complex associates with p53, and its activity is required for gene induction and RNA Polymerase II recruitment by p53. Moreover, cell proliferation is diminished in response to Nutlin alone but partially re-established by co-treatment with Nutlin and a CDK4 inhibitor.

We show that the complexes of CDK4 and Cyclin D1 on the one hand, and MDM2 and p53 on the other hand, associate with each other. These findings are in line with a previous report that MDMX, another close binding partner of MDM2 and p53, is phosphorylated by CDK4^37^. According to this report, CDK4-mediated phosphorylation of MDMX stabilizes its interaction with MDM2 to antagonize p53. This type of regulation would be different from the support of p53 activity by CDK4 reported here. However, since CDK4 needs to associate with MDMX at least temporarily to phosphorylate it, it is conceivable that this interaction further enables the association between the two complexes. This notion is further strengthened by a recent report on a physical association between Cyclin D1 and MDM2^38^. p53 is a phosphoprotein that undergoes numerous posttranslational modifications. Given that we observed an interaction of CDK4–Cyclin D1 with p53, one might ask whether p53 might be a substrate for the kinase CDK4. However, in vitro kinase assays did not reveal any such direct phosphorylation^39^.

How could CDK4 and/or Cyclin D1 assist in the recruitment of RNA Polymerase II to p53-responsive promoters? Previous studies revealed that Cyclin D1^40,41^ as well as the CDK4-related kinase CDK6^42^ can associate with target genes and function as transcriptional regulators. Interestingly, one of the genome-wide ChIP analyses revealed that Cyclin D1 binds with some preference to sites that also associate with p53, among other transcription factors^43^. These observations further support the idea that CDK4/Cyclin D1 might act as a co-factor for p53 in target gene activation.

A previous report is in seeming contradiction with our findings, claiming that Palbociclib cooperates with the MDM2 antagonist Idasanutlin (RG-7388) in killing liposarcoma cells^17^. Despite testing numerous conditions, we were unable to observe a similar synergy. One of the differences might be that in their study, the authors mostly used Idasanutlin concentrations that had little effect on tumor cell growth on their own, thus precluding any possible antagonism to begin with. While we can never rule out that the drugs might cooperate more favorably under clinical conditions, our results do give rise to caution when treating patients. We suggest that the combination of inhibitors to CDK4 and MDM2 in the clinic should be either avoided entirely or otherwise should be used only after carefully balancing the potential benefits with the antagonism reported here.

Another previous report argued that the cytotoxic effects of Nutlin on p53-proficient cells might depend on the ability of MDM2 to degrade the retinoblastoma protein Rb^27^. In cells where accumulated MDM2 leads to the degradation of hypophosphorylated Rb, apoptosis can be induced, but when Rb remains, the cells merely arrest. Such a scenario would argue against the efficacy of combining CDK4 inhibition with Nutlin, in line with our observations. CDK4 inhibition can be expected to increase the accumulation of hypophosphorylated Rb, since CDK4 is a key driver of Rb phosphorylation^25^. This, perhaps in addition to the diminished p53 activity reported here, would then result in decreased apoptosis^27^.

Some conventional chemotherapeutic drugs depend on the activation of p53 for their efficacy. Comparing p53-proficient and -deficient HCT116 cells, such a dependency was found in particular for 5-fluorouracil (5-FU)^44^. On the other hand, the same report showed that p53-deficient cells displayed a higher sensitivity towards topoisomerase inhibitors such as Adriamycin. In analogy, the combination of CDK4 inhibitors and the resulting p53 attenuation would be counterproductive for 5-FU but might be beneficial for Adriamycin. However, given the additional cell cycle regulatory functions of CDK4 inhibition, each combination remains to be tested individually.

What combinations could be more promising for cancer treatment? Our previous work suggests that the phosphatase PPM1D/Wip1 might represent a suitable target of drugs that synergize with MDM2 antagonists^19,45^. Wip1 dephosphorylates p53, thereby compromising its activity as a transcription factor. Interfering with this dephosphorylation enhances activating p53 modifications. When this is combined with MDM2 inhibitors, both stability and activity of p53 are increased, leading to pronounced cell death. The DNA damaging drug Trabectedin, currently used in second line for treating soft tissue sarcoma, was also reported to synergize with the MDM2 inhibitor RG7112^46^, perhaps as a result of p53 accumulation (through MDM2 inhibition) and activating p53 modifications (through DNA damage response). In preclinical investigations and cell culture, MDM2 antagonists also cooperated efficiently with mitogen-activated protein kinase kinase (MEK) or phosphatidylinositol-3 kinase (PI3K) inhibitors, BH3 mimetics, BCR-ABL antagonists, and histone deacetylase inhibitors^47^. For neuroblastoma cells, MDM2 inhibitors cooperate with anaplastic lymphoma kinase (ALK) inhibition^48^. In the case of CDK4 inhibitors, combination partners are less obvious. In breast cancer treatment, the CDK4/6 inhibitor Palbociclib is often combined with Letrozole, an inhibitor of aromatase in estrogen production, but this only makes sense when treating estrogen-dependent tumors^1^. CDK4 inhibition was reported to antagonize drugs that require entry into mitosis for their efficacy, such as taxanes^49^, probably due to inhibited cell cycle progression. On the other hand, CDK4 inhibitors were found to cooperate with inhibitors of signaling kinases, such as MEK and PI3K^49,50^. The underlying mechanisms for this cooperation, however, remain to be elucidated.

On the other hand, the drug antagonism reported here may be used in a beneficial way. In cases where p53 is mutated in a tumor, p53 activation by chemotherapeutics can be considered irrelevant. In such a scenario, it may be beneficial for normal cells if p53-induced cell death is attenuated. Thus, combining CDK4 inhibitors with DNA damaging chemotherapy might turn out to protect non-cancerous tissue in a patient, giving rise to a potential strategy for avoiding undesired general toxicities.

Under physiological conditions, the CDK4–Cyclin D1 complex is active in cycling cells and stem cells, whereas it is inactive in post-mitotic and terminally differentiated cells^51^. The positive impact of the CDK4–Cyclin D1 complex on p53 activity may thus constitute a greater sensitivity of cycling cells towards p53 activation, as compared to resting cells. Physiologically, this would make sense, since cycling cells are at higher risk for giving rise to cancer, whereas post-mitotic cells are unlikely to resume proliferation anyway, and preserving them despite genotoxic stress might help the organism to survive. Thus, the dependence of p53 activity on CDK4 appears as a mechanism to channel tumor suppression on cancer-prone, proliferating cells, while sparing differentiated cells despite DNA damage.

## Electronic supplementary material

Legends, Supplementary Material

All supplemental figures

Supplemental Table S1

Supplemental Table S2

## References

[1] O’Leary B, Finn RS, Turner NC (2016). Treating cancer with selective CDK4/6 inhibitors. Nat. Rev. Clin. Oncol..

[2] Vassilev LT (2004). In vivo activation of the p53 pathway by small-molecule antagonists of MDM2. Science.

[3] Khoo KH, Verma CS, Lane DP (2014). Drugging the p53 pathway: understanding the route to clinical efficacy. Nat. Rev. Drug Discov..

[4] Nguyen D, Liao W, Zeng SX, Lu H (2017). Reviving the guardian of the genome: small molecule activators of p53. Pharmacol. Ther..

[5] Muller CR, Paulsen EB, Noordhuis P, Pedeutour F, Saeter G, Myklebost O (2007). Potential for treatment of liposarcomas with the MDM2 antagonist Nutlin-3A. Int. J. Cancer.

[6] Verreault M (2016). Preclinical efficacy of the MDM2 inhibitor RG7112 in MDM2-amplified and TP53 wild-type glioblastomas. Clin. Cancer Res..

[7] Zhao Y, Aguilar A, Bernard D, Wang S (2015). Small-molecule inhibitors of the MDM2-p53 protein-protein interaction (MDM2 Inhibitors) in clinical trials for cancer treatment. J. Med. Chem..

[8] Wang S, Zhao Y, Aguilar A, Bernard D, Yang CY (2017). Targeting the MDM2-p53 protein-protein interaction for new cancer therapy: progress and challenges. Cold Spring Harb. Perspect. Med.

[9] Pilotti S (1998). Molecular abnormalities in liposarcoma: role of MDM2 and CDK4-containing amplicons at 12q13-22. J. Pathol..

[10] Bill KL (2016). SAR405838: a novel and potent inhibitor of the MDM2:p53 axis for the treatment of dedifferentiated liposarcoma. Clin. Cancer Res..

[11] Ohnstad HO (2013). Correlation of TP53 and MDM2 genotypes with response to therapy in sarcoma. Cancer.

[12] Burgess A, Chia KM, Haupt S, Thomas D, Haupt Y, Lim E (2016). Clinical overview of MDM2/X-targeted therapies. Front. Oncol..

[13] Ray-Coquard I (2012). Effect of the MDM2 antagonist RG7112 on the P53 pathway in patients with MDM2-amplified, well-differentiated or dedifferentiated liposarcoma: an exploratory proof-of-mechanism study. Lancet Oncol..

[14] Muthusamy V (2006). Amplification of CDK4 and MDM2 in malignant melanoma. Genes Chromosomes Cancer.

[15] Wunder JS, Eppert K, Burrow SR, Gokgoz N, Bell RS, Andrulis IL (1999). Co-amplification and overexpression of CDK4, SAS and MDM2 occurs frequently in human parosteal osteosarcomas. Oncogene.

[16] Berner JM, Forus A, Elkahloun A, Meltzer PS, Fodstad O, Myklebost O (1996). Separate amplified regions encompassing CDK4 and MDM2 in human sarcomas. Genes Chromosomes Cancer.

[17] Laroche-Clary A, Chaire V, Algeo MP, Derieppe MA, Loarer FL, Italiano A (2017). Combined targeting of MDM2 and CDK4 is synergistic in dedifferentiated liposarcomas. J. Hematol. Oncol..

[18] Oliner JD, Pietenpol JA, Thiagalingam S, Gyuris J, Kinzler KW, Vogelstein B (1993). Oncoprotein MDM2 conceals the activation domain of tumour suppressor p53. Nature.

[19] Sriraman A, Radovanovic M, Wienken M, Najafova Z, Li Y, Dobbelstein M (2016). Cooperation of Nutlin-3a and a Wip1 inhibitor to induce p53 activity. Oncotarget.

[20] Denissov S (2007). Identification of novel functional TBP-binding sites and general factor repertoires. EMBO J..

[21] Wienken M (2016). MDM2 associates with polycomb repressor complex 2 and enhances stemness-promoting chromatin modifications independent of p53. Mol. Cell.

[22] Langmead B, Salzberg SL (2012). Fast gapped-read alignment with Bowtie 2. Nat. Methods.

[23] Anders S, Pyl PT, Huber W (2015). HTSeq--a Python framework to work with high-throughput sequencing data. Bioinformatics.

[24] Love MI, Huber W, Anders S (2014). Moderated estimation of fold change and dispersion for RNA-seq data with DESeq2. Genome Biol..

[25] Kato J, Matsushime H, Hiebert SW, Ewen ME, Sherr CJ (1993). Direct binding of cyclin D to the retinoblastoma gene product (pRb) and pRb phosphorylation by the cyclin D-dependent kinase CDK4. Genes Dev..

[26] Tovar C (2006). Small-molecule MDM2 antagonists reveal aberrant p53 signaling in cancer: implications for therapy. Proc. Natl. Acad. Sci. USA.

[27] Du W, Wu J, Walsh EM, Zhang Y, Chen CY, Xiao ZX (2009). Nutlin-3 affects expression and function of retinoblastoma protein: role of retinoblastoma protein in cellular response to nutlin-3. J. Biol. Chem..

[28] Italiano A (2009). Variability of origin for the neocentromeric sequences in analphoid supernumerary marker chromosomes of well-differentiated liposarcomas. Cancer Lett..

[29] Persson F (2008). Characterization of the 12q amplicons by high-resolution, oligonucleotide array CGH and expression analyses of a novel liposarcoma cell line. Cancer Lett..

[30] Bieging KT, Mello SS, Attardi LD (2014). Unravelling mechanisms of p53-mediated tumour suppression. Nat. Rev. Cancer.

[31] D’Andrea AD, Haseltine WA (1978). Sequence specific cleavage of DNA by the antitumor antibiotics neocarzinostatin and bleomycin. Proc. Natl. Acad. Sci. USA.

[32] D’Orazi G (2002). Homeodomain-interacting protein kinase-2 phosphorylates p53 at Ser 46 and mediates apoptosis. Nat. Cell Biol..

[33] Hofmann TG (2002). Regulation of p53 activity by its interaction with homeodomain-interacting protein kinase-2. Nat. Cell Biol..

[34] Hallett ST (2017). Differential regulation of G1 CDK complexes by the Hsp90-Cdc37 chaperone system. Cell Rep..

[35] Barlev NA (2001). Acetylation of p53 activates transcription through recruitment of coactivators/histone acetyltransferases. Mol. Cell.

[36] Sakaguchi K (1998). DNA damage activates p53 through a phosphorylation-acetylation cascade. Genes Dev..

[37] Gerarduzzi C, de Polo A, Liu XS, El Kharbili M, Little JB, Yuan ZM (2016). Human epidermal growth factor receptor 4 (Her4) suppresses p53 protein via targeting the MDMX-MDM2 protein complex: implication of a novel MDMX SER-314 phosphosite. J. Biol. Chem..

[38] Yang P (2016). Downregulation of cyclin D1 sensitizes cancer cells to MDM2 antagonist Nutlin-3. Oncotarget.

[39] Wang Y, Prives C (1995). Increased and altered DNA binding of human p53 by S and G2/M but not G1 cyclin-dependent kinases. Nature.

[40] Pestell RG (2013). New roles of cyclin D1. Am. J. Pathol..

[41] Bienvenu F (2010). Transcriptional role of cyclin D1 in development revealed by a genetic-proteomic screen. Nature.

[42] Tigan AS, Bellutti F, Kollmann K, Tebb G, Sexl V (2016). CDK6-a review of the past and a glimpse into the future: from cell-cycle control to transcriptional regulation. Oncogene.

[43] Casimiro MC (2012). ChIP sequencing of cyclin D1 reveals a transcriptional role in chromosomal instability in mice. J. Clin. Invest..

[44] Bunz F (1999). Disruption of p53 in human cancer cells alters the responses to therapeutic agents. J. Clin. Invest..

[45] Sriraman A, Li Y, Dobbelstein M (2016). Fortifying p53 - beyond Mdm2 inhibitors. Aging.

[46] Obrador-Hevia A (2015). RG7112, a small-molecule inhibitor of MDM2, enhances trabectedin response in soft tissue sarcomas. Cancer Invest..

[47] Saiki AY (2014). MDM2 antagonists synergize broadly and robustly with compounds targeting fundamental oncogenic signaling pathways. Oncotarget.

[48] Wang HQ (2017). Combined ALK and MDM2 inhibition increases antitumor activity and overcomes resistance in human ALK mutant neuroblastoma cell lines and xenograft models. Elife.

[49] Franco J, Witkiewicz AK, Knudsen ES (2014). CDK4/6 inhibitors have potent activity in combination with pathway selective therapeutic agents in models of pancreatic cancer. Oncotarget.

[50] Laroche A, Chaire V, Algeo MP, Karanian M, Fourneaux B, Italiano A (2017). MDM2 antagonists synergize with PI3K/mTOR inhibition in well-differentiated/dedifferentiated liposarcomas. Oncotarget.

[51] Latella L (2001). Reconstitution of cyclin D1-associated kinase activity drives terminally differentiated cells into the cell cycle. Mol. Cell. Biol..

[52] Liberzon A, Birger C, Thorvaldsdottir H, Ghandi M, Mesirov JP, Tamayo P (2015). The Molecular Signatures Database (MSigDB) hallmark gene set collection. Cell Syst..

