# Supplementary material for: CDK4 inhibition diminishes p53 activation by MDM2 antagonists
Source: Cell Death Dis. 2018 Sep 11;9(9):918. doi: 10.1038/s41419-018-0968-0 (PMC6133967; doi:10.1038/s41419-018-0968-0)
Supplement: Supplementary file 1 — Legends, Supplementary Material [file 41419_2018_968_MOESM1_ESM.docx]

**LEGENDS TO SUPPLEMENTARY MATERIAL**

**Supplementary Figure S1. The combination of Nutlin with PD0332991 revealed antagonistic effects with regard to cell viability**

1. Snapshot of the cBioportal dataset from the cancer cell line collections CCLE and NCI-60, with the cell line SJSA containing amplifications of CDK4 and MDM2, according to www.cbioportal.org.
2. SJSA, CRL3043, CRL3044 and GOT-3 cells were treated with Nutlin at the indicated concentrations for 24 hours. Immunoblot analysis confirmed the expected accumulation of p53, p21 and MDM2.
3. To confirm the efficacy of PD0332991, SJSA, CRL3043, CRL3044 and GOT-3 cells were treated with it at the indicated concentrations for 24 hours. Reduced phosphorylation on Rb at S807/811 was detected by immunoblot analysis.
4. Combinatorial treatment with Nutlin and PD0332991 in SJSA cells with increasing concentrations of the two drugs revealed antagonism with respect to cell viability, by luciferase assay to determine ATP levels 72 hours post-treatment.
5. Varying drug concentrations were used to treat CRL3043 cells. Cell viability assays (ATP-luciferase) were performed after 72 hours to reveal antagonistic cytotoxicity of inhibitors to MDM2 and CDK4/6.
6. CRL3044 cells were treated with increasing concentrations of Nutlin and PD0332991, alone or in combination, followed by viability assays as in e.
7. Viability analyses as in e and f, using another liposarcoma cell line, GOT-3, with similar results.

**Supplementary Figure S2. Alternate CDK4/6 inhibitors decrease p53 target gene expression in various sarcoma cell lines upon treatment with MDM2 antagonists**

1. Treatment scheme.
2. CRL3043 cells were treated with the indicated drugs and harvested for immunoblot analysis. Upon Nutlin treatment, the p53 target genes p21 and MDM2 were induced. This induction was diminished upon co-treatment with PD0332991. β-actin serves as loading control.
3. CRL3044 cells were treated as in B.
4. SJSA cells were treated with RG7388, an MDM2 antagonist distinct from Nutlin, alone or in combination with various FDA-approved CDK4/6 inhibitors, i.e. LEE011 (Ribociclib), LY2835219 (Abemaciclib) and PD0332991 (Palbociclib). Again, we observed that RG7388 treatment accumulated p53 and its target genes while treatment with CDK4/6 inhibitors decreases the activation.
5. SJSA cells were treated with various CDK4/6 inhibitors for 24 hours to confirm that they reduce the levels of phosphorylated (807/811) pRb.
6. SJSA cells were treated with Nutlin and the HIPK2 inhibitor A-64 as indicated.
7. SJSA cells were harvested for immunoblot analysis after treatment as in F. Upon Nutlin treatment, p53 as well as MDM2 and p21 proteins accumulated. Their levels remained similar upon co-treatment with the HIPK2 inhibitor A-64.

**Supplementary Figure S3. Impaired complex formation of CDK4 with cyclin D1 upon combined inhibition of MDM2 and CDK4/6**

Band intensities from Fig. 5c, corresponding to CDK4 and cyclin D1, upon treatment with Nutlin alone or Nutlin with the CDK4/6 inhibitor and precipitation with antibodies to Cyclin D1 and CDK4, respectively. The intensities obtained were further normalized to Nutlin treatment. The reduction shows diminished interaction of the two proteins upon CDK4 inhibitor treatment. Mean of three biological experiments.

**Supplementary Figure S4. Alternate RNA polymerase II antibodies indicate decreased occupancy of p53-responsive promoters upon combined treatment**

1. SJSA cells were treated with Nutlin and PD0332991 as indicated in the scheme.
2. The indicated antibody to RNA polymerase antibody (MBL life sciences) was used for chromatin immunoprecipitation (ChIP). We observed that with Nutlin treatment, there is an enhanced occupancy of RNA polymerase II at the TSSs of p53 target genes which is decreased when the cells are co-treated with PD0332991. MyoD served as a negative site, IgG as a negative ChIP control. Mean of two independent experiments.
3. The antibody A-10 to RNA polymerase enzyme (Santa Cruz) was utilized for ChIP as in B, with similar results.

**Supplementary Table 1 related to Figure 4**

SJSA cells were treated with Nutlin, PD0332991 and their combinations as mentioned. Global gene expression was analyzed via next generation RNA sequencing. DESeq2 was carried out for Nutlin vs DMSO sample. With the gene list obtained after DESeq2, Z-score analysis was performed for all the conditions. Base mean values, log2 fold change (FC) and adjusted p values are shown for each gene.

**Supplementary Table 2 related to Figure 4**

Gene set enrichment analysis (GSEA) from C2 curated gene sets (provided by the Molecular Signatures Database (MSigDB) v5.0) was performed using variance stabilized SJSA RNASeq data from Nutlin and Nutlin + PD0332991 treated samples. The threshold of significant enrichment (q≤0.25) was implied according to the GSEA standards (http://www.broadinstitute.org/gsea/doc/GSEAUserGuideFrame.html).
