# Supplementary material for: CDK4 inhibition diminishes p53 activation by MDM2 antagonists
Source: Cell Death Dis. 2018 Sep 11;9(9):918. doi: 10.1038/s41419-018-0968-0 (PMC6133967; doi:10.1038/s41419-018-0968-0)
Supplement: Supplementary file 2 — All supplemental figures [file 41419_2018_968_MOESM2_ESM.pptx]

## Slide 1
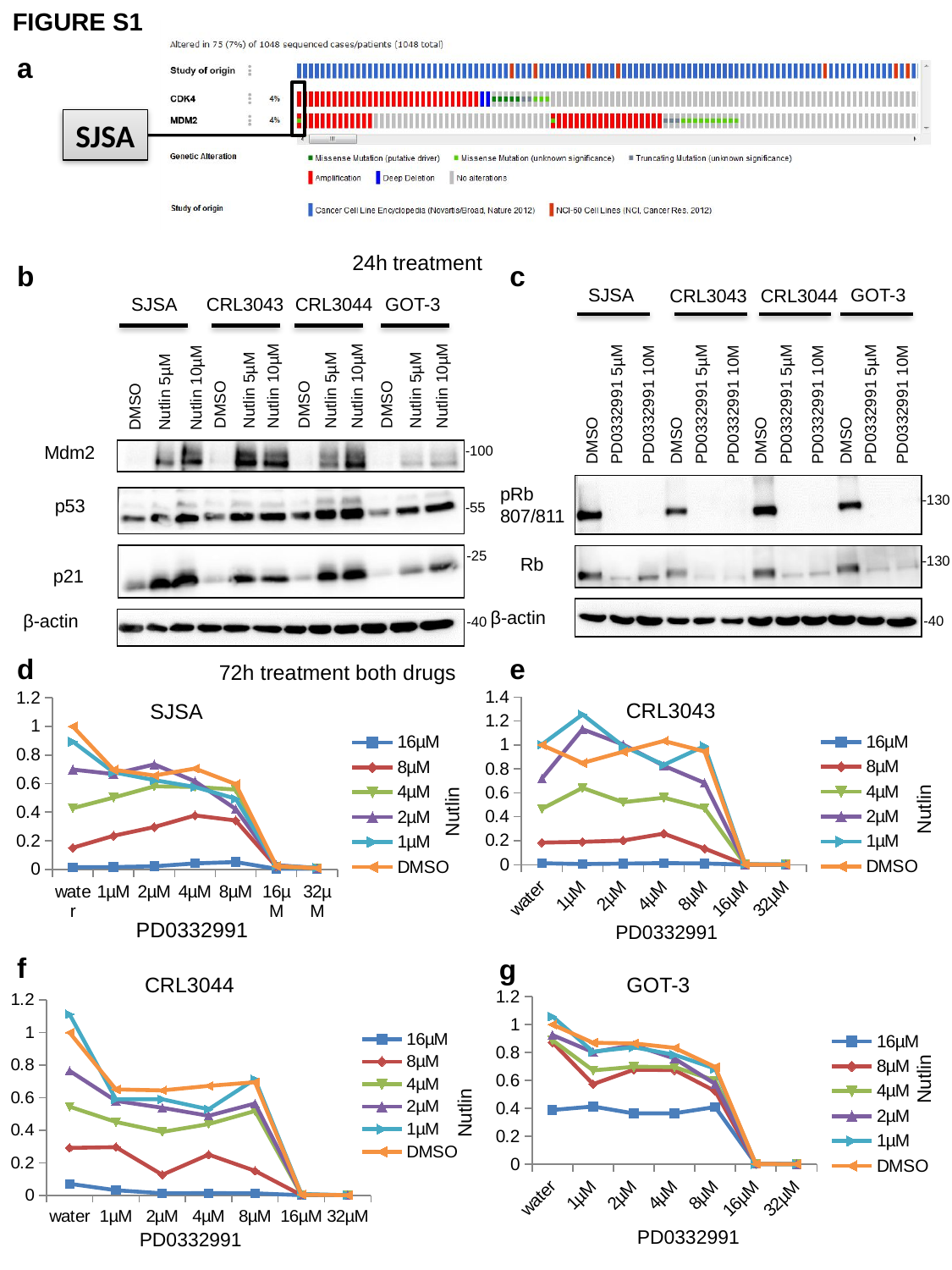

FIGURE S1
a
SJSA
24h treatment
b
c
SJSA
GOT-3
CRL3043
CRL3044
SJSA
CRL3043
CRL3044
GOT-3
Nutlin 5µM
Nutlin 5µM
Nutlin 5µM
Nutlin 5µM
Nutlin 10µM
Nutlin 10µM
Nutlin 10µM
Nutlin 10µM
PD0332991 10M
PD0332991 10M
PD0332991 10M
PD0332991 10M
DMSO
DMSO
DMSO
DMSO
PD0332991 5µM
PD0332991 5µM
PD0332991 5µM
PD0332991 5µM
DMSO
DMSO
DMSO
DMSO
Mdm2
-100
pRb 807/811
-130
p53
-55
-25
-130
Rb
p21
β-actin
β-actin
-40
-40
d
e
72h treatment both drugs
### Chart
| Category | 16µM | 8µM | 4µM | 2µM | 1µM | DMSO |
|---|---|---|---|---|---|---|
| water | 0.011701444114495387 | 0.1842566288604295 | 0.46588806861305965 | 0.7180886653816019 | 1.0032116403575708 | 1.0 |
| 1µM | 0.005048322724909315 | 0.19061861872905259 | 0.6412718259258238 | 1.130777300730689 | 1.2544438417511203 | 0.8493154323200935 |
| 2µM | 0.009147658842890108 | 0.20185833846644172 | 0.5218067724342375 | 1.0031503495110523 | 0.990238411177816 | 0.9435879048643481 |
| 4µM | 0.013039627596816553 | 0.2588598472427802 | 0.5596804295262524 | 0.825814478736673 | 0.8338915907937062 | 1.0332145312424983 |
| 8µM | 0.009975085270890218 | 0.13455383839033894 | 0.47139913722918386 | 0.6825992222191577 | 0.9909013738343248 | 0.9454736199089013 |
| 16µM | 0.0006384463179013197 | 0.0006997371644198464 | 0.00113694520291867 | 0.0016364656020446627 | 0.004385360068400585 | 0.0014168400686866086 |
| 32µM | 0.0003462932828296758 | 0.0004198422986519078 | 0.0005475315622321718 | 0.0005291443082766137 | 0.00048624071571364507 | 0.0010092559393384062 |
### Chart
| Category | 16µM | 8µM | 4µM | 2µM | 1µM | DMSO |
|---|---|---|---|---|---|---|
| water | 0.01481995572334037 | 0.15101626034603663 | 0.4276542305147951 | 0.6985802769412103 | 0.8940672344596235 | 1.0 |
| 1µM | 0.015765775929056265 | 0.23473578855503402 | 0.5023722217659582 | 0.6669413275597388 | 0.6795366202992149 | 0.697360096675821 |
| 2µM | 0.02104269457678607 | 0.2957141168178204 | 0.5810350413751215 | 0.7344429272413366 | 0.62456533334296 | 0.656451567778216 |
| 4µM | 0.041505081527355235 | 0.37698245449368384 | 0.5772517605522579 | 0.6158065689379287 | 0.5762418628326051 | 0.7053129284055619 |
| 8µM | 0.05102104359707698 | 0.3416252043034819 | 0.5583263314359771 | 0.42215168681799187 | 0.4944148775352359 | 0.596773742298289 |
| 16µM | 0.003212900698805902 | 0.018447104012245124 | 0.019081561650239658 | 0.029355623884848196 | 0.025083187955593382 | 0.023068807517465637 |
| 32µM | 0.005137031117304268 | 0.006335551377982425 | 0.007216391569565167 | 0.008822841918968117 | 0.0101774947136051 | 0.008920311940167847 |CRL3043
SJSA
Nutlin
Nutlin
PD0332991
PD0332991
f
g
GOT-3
CRL3044
### Chart
| Category | 16µM | 8µM | 4µM | 2µM | 1µM | DMSO |
|---|---|---|---|---|---|---|
| water | 0.3877748126782478 | 0.8728693852592654 | 0.8879330253643545 | 0.9246321641220506 | 1.0577017866658762 | 1.0 |
| 1µM | 0.4129275275596848 | 0.573186293197406 | 0.6716304317103722 | 0.8018108631105234 | 0.8058823407756489 | 0.8691343249833342 |
| 2µM | 0.3629261143308982 | 0.6766256784359901 | 0.6973498168662307 | 0.8529125266531502 | 0.8371719154902973 | 0.8646809308558858 |
| 4µM | 0.3642683369881958 | 0.6741177143747428 | 0.6958593775313168 | 0.7564593172795139 | 0.7834306163263083 | 0.8330508024726678 |
| 8µM | 0.4092379663568823 | 0.5235867884479232 | 0.5989918508402506 | 0.574832532388791 | 0.6841619794578578 | 0.6960399950640399 |
| 16µM | 0.0020316025531873826 | 0.0013422226572975342 | 0.0010382061232101112 | 0.0014814773962672837 | 0.001325677539796178 | 0.0017482674159766548 |
| 32µM | 0.00043913499368183326 | 0.0004515438318078505 | 0.00035296250669560226 | 0.0004101810380544596 | 0.00043913499368183326 | 0.0004556801111831896 |
### Chart
| Category | 16µM | 8µM | 4µM | 2µM | 1µM | DMSO |
|---|---|---|---|---|---|---|
| water | 0.07055859705395431 | 0.2911681582863725 | 0.5446824202822601 | 0.7648655779572864 | 1.1123654513512433 | 1.0 |
| 1µM | 0.030883760543625363 | 0.2958211052413783 | 0.4506699488175835 | 0.5800890243332618 | 0.5898876325901022 | 0.6507108544606877 |
| 2µM | 0.011904325561913993 | 0.12583073849235005 | 0.3892770697010335 | 0.5373708863178458 | 0.59019260626304 | 0.6446236918657926 |
| 4µM | 0.013192209797265254 | 0.2497843500381497 | 0.4373845681642739 | 0.4883655341697819 | 0.5284284426841153 | 0.6723108246907805 |
| 8µM | 0.01197427365203735 | 0.14987301623719004 | 0.5180632548972757 | 0.5639749429153637 | 0.7133429339418631 | 0.6949533012560718 |
| 16µM | 0.0003964657748191912 | 0.0011477082627440523 | 0.00254051463328035 | 0.005367536643705973 | 0.007887906227030795 | 0.004044678363293033 |
| 32µM | 0.00031308765139214886 | 0.00031644515971807004 | 0.00031644515971807004 | 0.0004048595456339941 | 0.00031308765139214886 | 0.00036149172975751237 |Nutlin
Nutlin
PD0332991
PD0332991

## Slide 2
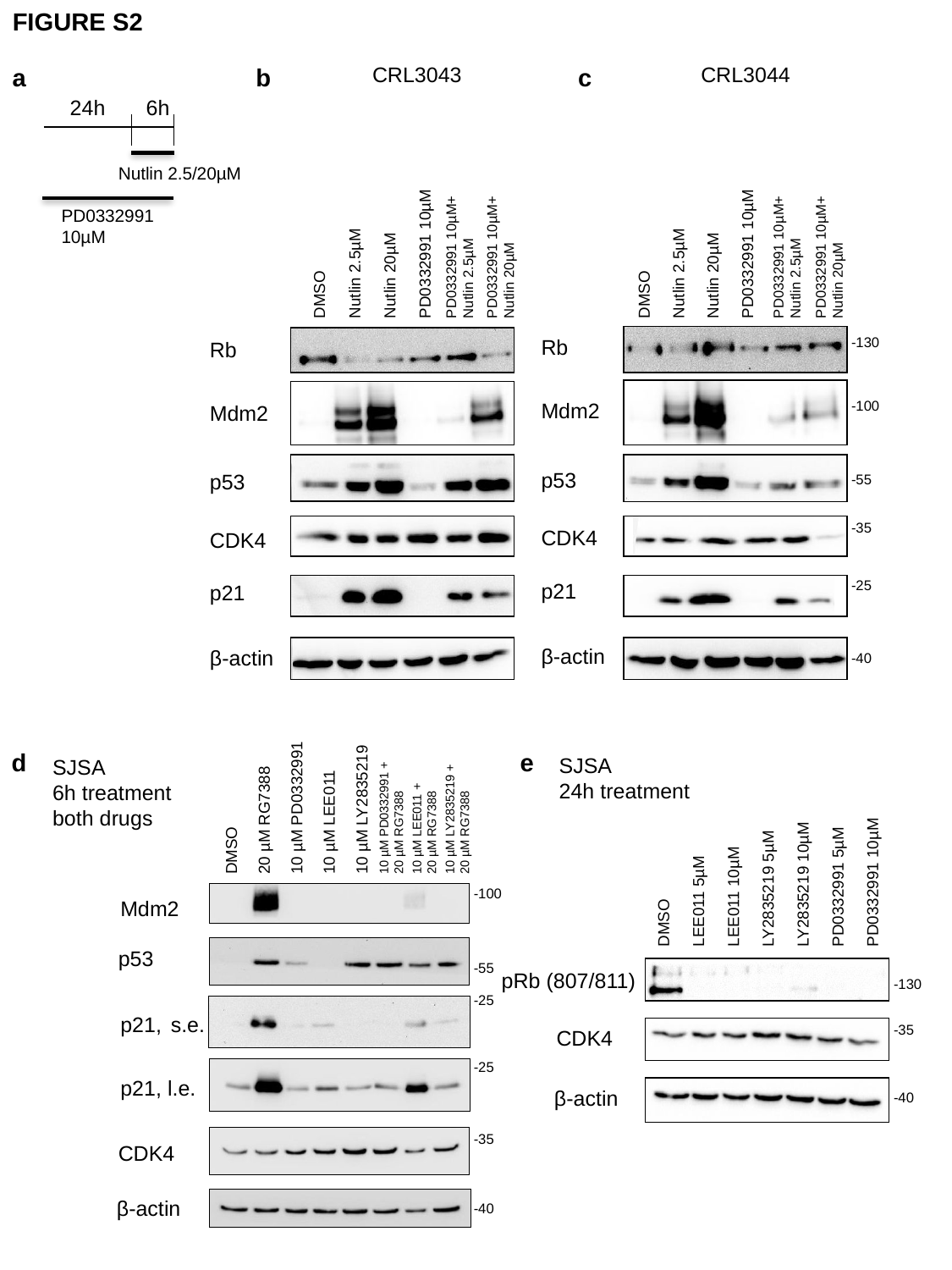

FIGURE S2
a
b
CRL3043
c
CRL3044
24h
6h
Nutlin 2.5/20µM
PD0332991
10µM
PD0332991 10µM+
Nutlin 2.5µM
PD0332991 10µM+
Nutlin 20µM
PD0332991 10µM+
Nutlin 2.5µM
PD0332991 10µM+
Nutlin 20µM
Nutlin 2.5µM
Nutlin 20µM
PD0332991 10µM
Nutlin 2.5µM
Nutlin 20µM
PD0332991 10µM
DMSO
DMSO
-130
Rb
Rb
-100
Mdm2
Mdm2
p53
p53
-55
-35
CDK4
CDK4
-25
p21
p21
β-actin
β-actin
-40
d
e
20 µM RG7388
10 µM LEE011
SJSA
24h treatment
SJSA
6h treatment both drugs
10 µM LY2835219 +
20 µM RG7388
10 µM PD0332991 +
20 µM RG7388
10 µM PD0332991
10 µM LEE011 +
20 µM RG7388
10 µM LY2835219
DMSO
DMSO
LEE011 5µM
LEE011 10µM
LY2835219 5µM
LY2835219 10µM
PD0332991 5µM
PD0332991 10µM
-100
Mdm2
p53
-55
pRb (807/811)
-130
-25
p21, s.e.
-35
CDK4
-25
p21, l.e.
β-actin
-40
-35
CDK4
β-actin
-40

## Slide 3
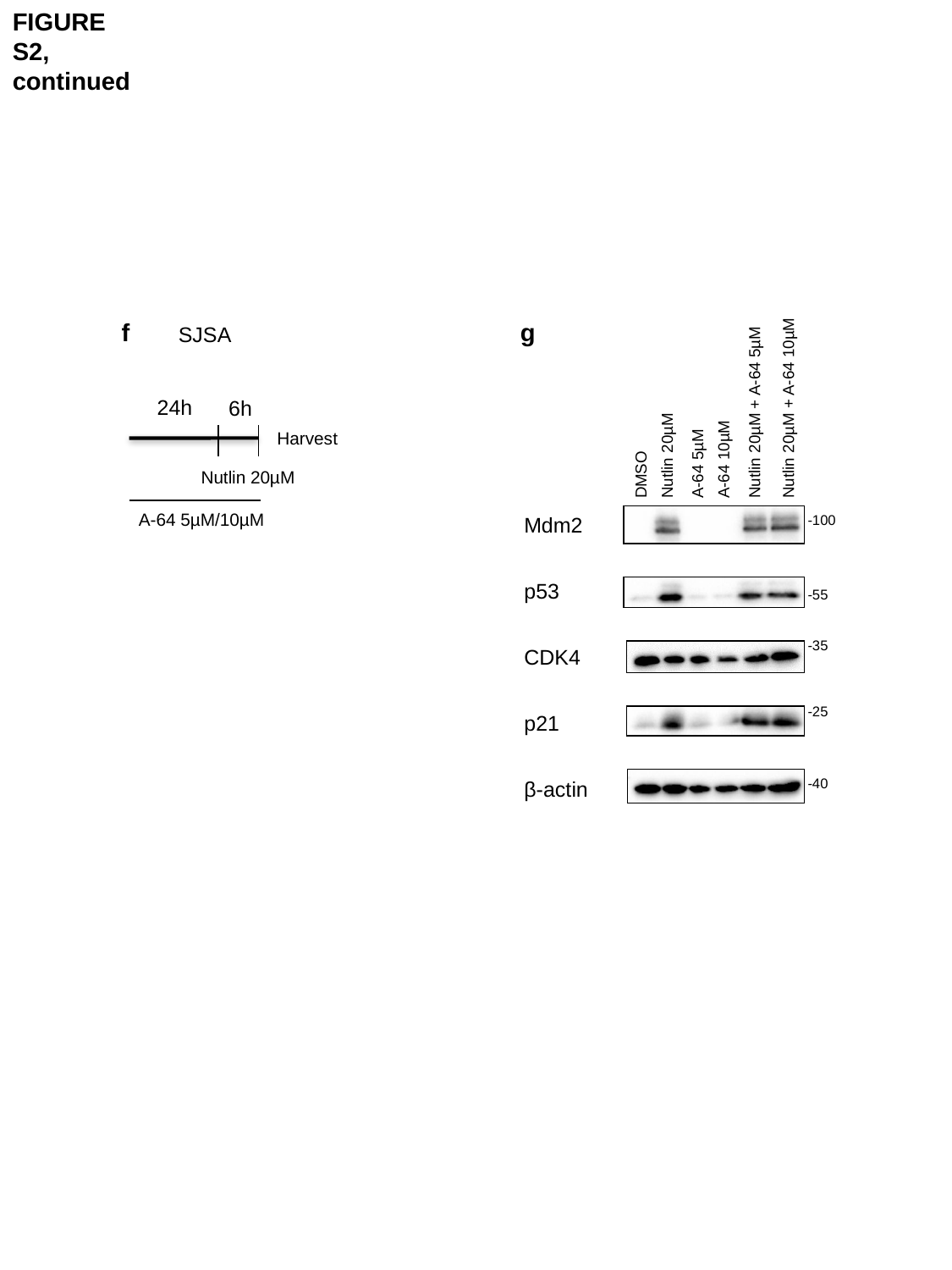

FIGURE S2, continued
f
g
SJSA
Nutlin 20µM + A-64 10µM
24h
6h
Nutlin 20µM
A-64 5µM
A-64 10µM
Nutlin 20µM + A-64 5µM
Harvest
DMSO
Nutlin 20µM
A-64 5µM/10µM
-100
Mdm2
p53
-55
-35
CDK4
-25
p21
-40
β-actin

## Slide 4
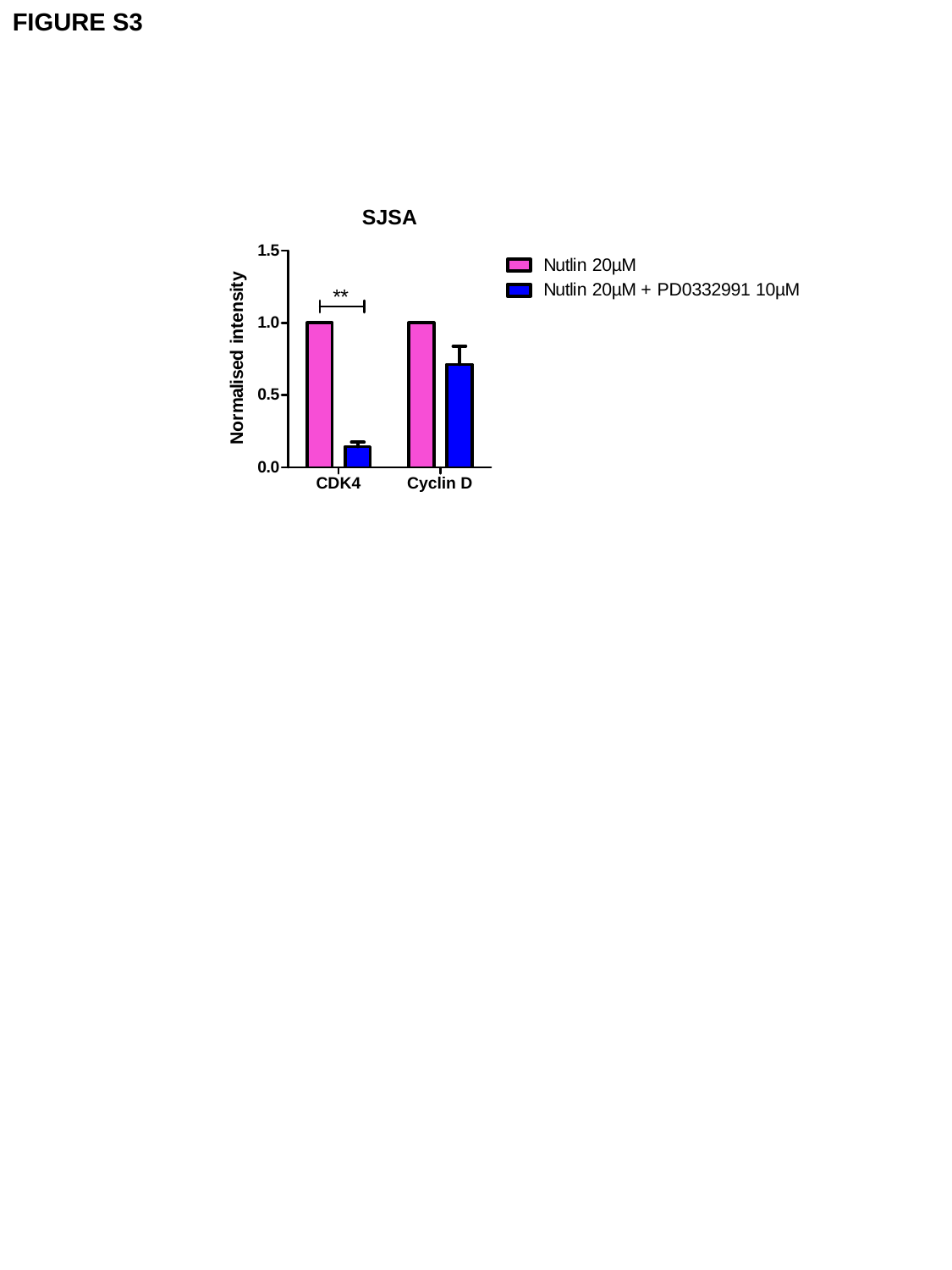

FIGURE S3

## Slide 5
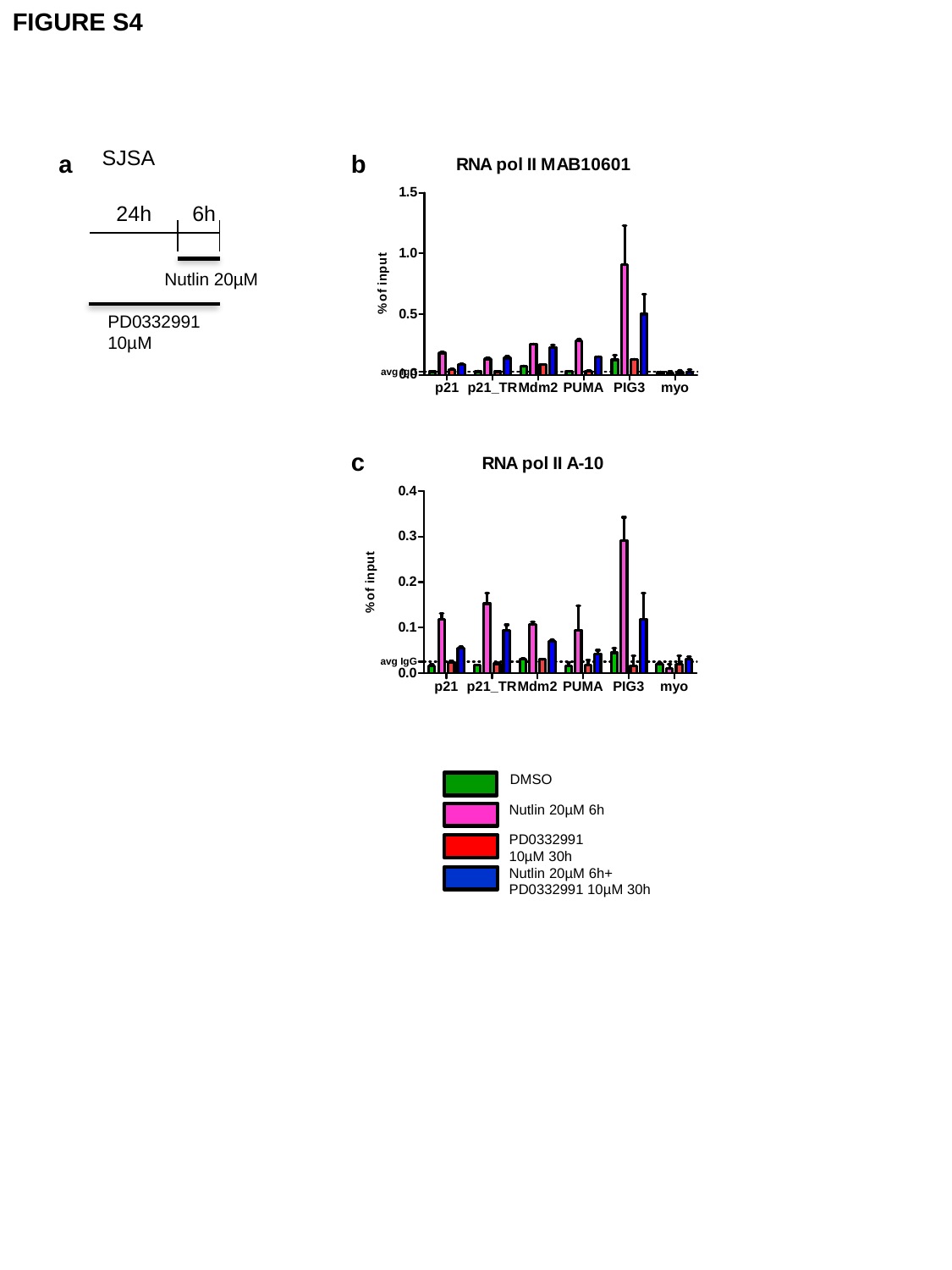

FIGURE S4
SJSA
a
b
24h
6h
Nutlin 20µM
PD0332991
10µM
c
DMSO
Nutlin 20µM 6h
PD0332991 10µM 30h
Nutlin 20µM 6h+
PD0332991 10µM 30h
